# Peroxidative Oxidation of Alkanes and Alcohols under Mild Conditions by Di- and Tetranuclear Copper (II) Complexes of Bis (2-Hydroxybenzylidene) Isophthalohydrazide

**DOI:** 10.3390/molecules23102699

**Published:** 2018-10-19

**Authors:** Manas Sutradhar, Elisabete C.B.A. Alegria, M. Fátima C. Guedes da Silva, Cai-Ming Liu, Armando J. L. Pombeiro

**Affiliations:** 1 Centro de Química Estrutural, Instituto Superior Técnico, Universidade de Lisboa, Av. Rovisco Pais, 1049-001 Lisboa, Portugal; pombeiro@tecnico.ulisboa.pt; 2 Chemical Engineering Departament, Instituto Superior de Engenharia de Lisboa, Instituto Politécnico de Lisboa, R. Conselheiro Emídio Navarro, 1, 1959-007 Lisboa, Portugal; 3 National Laboratory for Molecular Sciences, Center for Molecular Science, Institute of Chemistry, Chinese Academy of Sciences, Beijing 100190, China; cmliu@iccas.ac.cn

**Keywords:** Cu (II) complexes, microwave assisted oxidation of alcohols, oxidation of alkanes, solvent free process, magnetism

## Abstract

Bis(2-hydroxybenzylidene)isophthalohydrazide (H_4_L) has been used to synthesize the dinuclear [Cu_2_(1κ*NO*^2^:2κ*N′O′*^2^-H_2_L)(NO_3_)_2_(H_2_O)_2_] (**1**) and the tetranuclear [Cu_4_(μ-1κ*NO*^2^:2κ*N′O*^2^-H_2_L)_2_(μ-NO_3_)_2_(H_2_O)_4_]·2C_2_H_5_OH (**2**) complexes. The solvent plays an important role in determining the ligand behaviour in the syntheses of the complexes. An ethanol-acetonitrile mixture of solvents favours partials enolization in the case of **2**. Both complexes have been characterized by elemental analysis, infrared radiation (IR), single crystal X-ray crystallography and electrochemical methods. The variable temperature magnetic susceptibility measurements of **2** show strong antiferromagnetic coupling between the central nitrato-bridged Cu (II) ions. The catalytic activity of both **1** and **2** has been screened toward the solvent-free microwave-assisted oxidation of alcohols and the peroxidative oxidation of alkanes under mild conditions. Complex **1** exhibits the highest activity for both oxidation reactions, leading selectively to a maximum product yield of 99% (for the 1-phenylethanol oxidation after 1 h without any additive) and 13% (for the cyclohexane oxidation to cyclohexyl hydroperoxide, cyclohexanol and cyclohexanone after 3 h).

## 1. Introduction

The oxidation of alcohols to carbonyl-containing compounds [1,2,3] is one of the fundamental reactions in organic synthesis [4,5], with a great interest due to the application of carbonyl compounds in research and industrial manufacturing, e.g., in the production of new materials [6] and energy sources [7]. In view of their central role in synthetic chemistry and expected further applications, these reactions continue to attract great attention in order to develop environmentally benign processes [8,9] disclosing new efficient catalysts [10,11], substrates or oxidation agents which could successfully be used in the near future and make a difference in terms of efficiency, selectivity, economy and/or sustainability of the processes. The metal-catalyzed aerobic and peroxidative oxidations of alcohols, in particular of benzylic alcohols, are typical model reactions due to their importance and generality; inexpensive primary oxidants such as O_2_, H_2_O_2_ or *tert*-butylhydroperoxide (TBHP) and simple procedures are usually explored [1,2,3,4,5]. The accelerating effect of microwave (MW) irradiation in the synthesis of ketones from secondary alcohols with TBHP as an oxidant has been largely reported [12,13,14,15,16,17,18]; this technology is a useful alternative source in organic synthesis, with an environmentally friendly nature.

The mild oxidation of cycloalkanes by hydrogen peroxide to the corresponding alkyl hydroperoxides, alcohols and ketones, a highly significant reaction in terms of industrial interest, remains a challenge in modern catalysis [19,20,21,22,23,24].

It has been shown that copper (II) complexes are useful catalysts towards the MW assisted oxidation of alcohols and also the functionalization of inert alkanes into valuable organic products, using readily available and cheap oxidants [22,25,26,27,28]; however, such applications are still limited and the subject requires further exploration. Moreover, aroylhydrazone complexes are potentially important as oxidation catalyst and some of them also showed interesting magnetic properties [29,30,31,32].

Continuing our research on the syntheses of aroylhydrazone metal complexes and their application in catalysis, herein we report the syntheses and characterization of new dinuclear and tetranuclear Cu(II) complexes derived from the aroylhydrazone Schiff base bis(2-hydroxybenzylidene) isophthalohydrazide (H_4_L), their electrochemical behaviour and magnetic properties (**2**), as well as their catalytic activity in the solvent-free MW assisted peroxidative oxidation of alcohols and also in the oxidation of alkanes under mild conditions, towards the development of environmentally friendly catalytic systems.

## 2. Results and Discussion

### 2.1. Syntheses and Characterizations

**Scheme 1:**
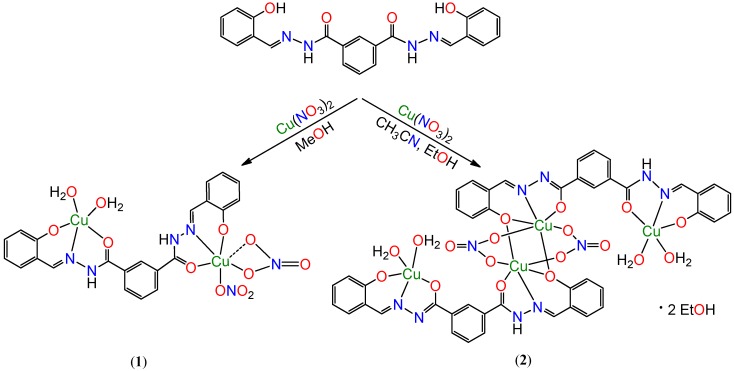
Syntheses of **1** and **2**.

The aroylhydrazone Schiff base bis(2-hydroxybenzylidene) isophthalohydrazide (H_4_L) has two potential tridentate coordination pockets which can bind two metal centres simultaneously. It has previously been observed [32,33,34] that the aroylhydrazone can act as ligand in two tautomeric (keto or enol) forms. At room temperature, deprotonation of the phenolic –OH group from the aroylhydrazone in the presence of a copper (II) source, in methanol, generates the H_2_L^2−^ species and leads to the stable dinuclear complex **1** (Scheme 1). When the reaction is carried out in a 2:1 acetonitrile-ethanol solvent mixture, part of the aroylhydrazone remains in the keto form, the remaining undergoing enolization and deprotonation during complexation. As a result, a HL^3^^−^ species is formed in solution and provides the tetranuclear complex **2** (Scheme 1). This compound **2** can be considered as a dimer of **1**, where the two central Cu (II) ions are bridged by two nitrate ions and one of the phenoxido groups of the organic ligand. The IR spectra of complexes **1** and **2** contain all the characteristic bands of the corresponding coordinated tridentate anionic ligand viz., 3476, 3024, 1608, 1254 and 1159 cm^−1^ for **1** and 3388, 3226, 2978, 1611, 1252 and 1068 cm^−1^ for **2**. The electrospray ionization mass spectrometry (ESI-MS) spectra of both compounds, in ethanol solution (see experimental section), display the molecular ion peaks at *m/z* = 672 [**1** + H]^+^ (100%) and at *m/z* = 1158 [**2** + H]^+^ (100%). 

### 2.2. General Description of the Crystal Structures

**Figure 1:**
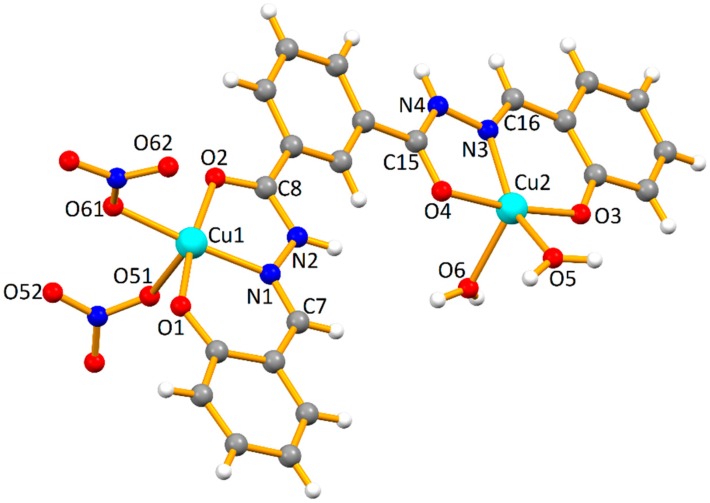
Molecular structure of **1** with partial atom numbering scheme. The O-atoms from solvent water molecules are omitted for clarity.

**Figure 2:**
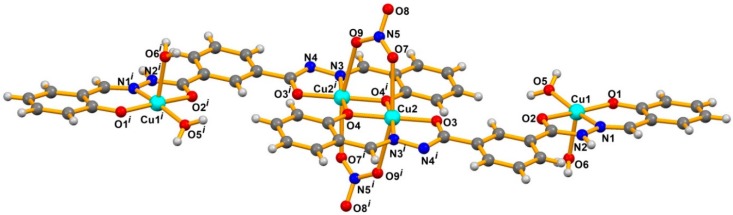
Molecular structure of **2** with partial atom numbering scheme. The ethanol solvent molecule is omitted for clarity. Symmetry operation to generate equivalent atoms (i): 3−x, −y, 1−z.

X-ray low quality (low diffracting) crystals of **1** and **2** were obtained upon slow evaporation of a methanolic (for **1**) or an acetonitrile-ethanol solution mixture (for **2**), at room temperature. Crystallographic data are summarized in Table 1, representative plots are displayed in Figure 1 and Figure 2, and selected dimensions are presented in Table 2. Cif files for Compound **1** and Compound **2** are available as Supporting Information for this paper.

The organic ligand in **1** and **2** is almost planar, behaves as a hexadentate chelator and acts as a NO_2_ donor for each metal cation in **1** and for the outer ones in **2**, while for the inner metals it behaves as an O_phenolate_-bridging species as well. The copper cations exhibit square pyramidal [Cu1: *τ*_5_ = 0.08 (**1**) and 0.06 (**2**)], distorted penta-coordinate (Cu2 in **1**, *τ*_5_ = 0.38) or distorted octahedral geometries (Cu2 in **2**; quadratic elongation = 1.078, angle variance = 77.60 º^2^). The relative orientation of one of the nitrate ligands of Cu1 in **1** caused a measured Cu-O_nitrate_ bond distance of 2.69(1) Å which is similar to those of 2.680 (7) and 2.709 (5) Å in **2**, well below the sum on the van der Waals radii of copper and oxygen (1.40 and 1.52 Å, respectively). Therefore, on this basis, the geometry of that cation in **1** may be thought as octahedral, although with a great distortion (quadratic elongation = 1.125, angle variance = 272.62 º^2^).

Compound **2** can be considered as a dimer of **1**, where dimerization took place at the level of the metals bearing nitrates, leading to a central four-membered dicopper metalacycle. This process affected not only the dimensions involving the central copper cations (Cu_(nitrate)_, see Table 2), but also the outer ones with water ligands bound to copper (Cu_(water)_, see Table 2), which is probably related to the observed change of geometry (see above the values of *τ*_5_ descriptor for such metals). Thus, from **1** to **2**, the Cu-O_phenolate_ increased in both types of metals, but the Cu-O_ketone_ increased only in the metal with water ligands, and decreased slightly in the other. Concerning the Cu-O_water_ lengths, they differ markedly in **2** with the apical much longer than the equatorial one; in **1** the dissimilarity is not so significant, but still the longer distance pertains to the water molecule more distant from the least-square plane of the molecule. An influence has also been perceived in the Cu···Cu lengths; in the molecule of **2** the distance between the Cu_(nitrate)_ and the Cu_(water)_ is more than 0.100 Å shorter than in **1**, but the shortest intermolecular distance between the metal cations is higher in the former. The effect on selected O-Cu-O and O-Cu-N angles in **1** and **2** (Table 2) is most probably related to the aforementioned differences in geometry.

Moreover, the enolate forms are more strongly coordinated to the metal centres with shorter M–O bond distances than the corresponding ones in the keto forms, as observed in other cases [32,33,34].

### 2.3. Electrochemical Properties

Cyclic voltammograms of **1** and **2** exhibit a first single-electron (per metal atom) irreversible reduction process (wave **I^red^**) at ^I^*E*_p_^red^ −0.27 or −0.43 V vs. SCE, for **1** or **2**, respectively, followed, at a lower potential, by a second single-electron (per metal atom) reduction (wave **II^red^**) at ^II^*E*_p_^red^ = −1.12 or −1.08 V vs. SCE, for **1** or **2**, respectively, which are believed to correspond to the Cu^II^ → Cu^I^ (wave **I^red^**) and Cu^I^ → Cu^0^ (wave **II^red^**) cathodic processes, in this order. Upon scan reversal following the first reduction wave, an irreversible oxidation at ^I^*E*_p_^ox^ −0.05 or 0.02 V vs. SCE, for **1** or **2**, respectively, was detected and assigned to the oxidation of a novel Cu(I) species. By reversing the direction of the potential scan after the formation of wave **II^red^**, an adsorption wave was formed at ^II^*E*_p_^ox^ 0.15 or 0.27 V vs. SCE for **1** or **2**, respectively.

Expectedly, no genuine anodic waves have been detected for any of the complexes. Bis(2-hydroxybenzylidene) isophthalohydrazide (H_4_L) was not redox active under our experimental conditions, and hence the cyclic voltammetric waves of **1** and **2** can be due to metal-based electron transfer processes.

### 2.4. Magnetic Properties

**Figure 3:**
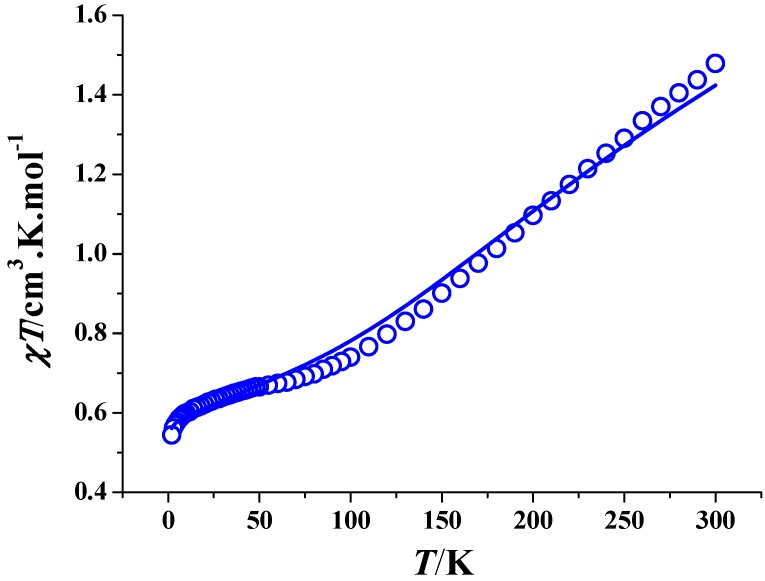
Plot of *χ_M_*T versus T for complex **2**. The solid line represents the best theoretical fitting.

The dc magnetic susceptibilities of complex **2** have been determined under 2000 Oe from 2 K to 300 K. As shown in Figure 3, the *χ*_M_*T* value at 300 K is 1.48 cm^3^ K mol^−1^, slightly smaller than the expected value for four noninteracting Cu^2+^ ions (*g* = 2.0). The *χ*_M_*T* product decreases with the decreased temperture until about 100 K, then drops slowly to about 10 K; below this temperature, *χ*_M_*T* further decreases. These results indicate that there are antiferromagnetic interactions between copper(II) ions.

A linear tetranuclear copper (II) magnetic coupling model was adopted to analyze the magnetic interaction [35]. Owing to the crystallographic inversion symmetry, four exchange coupling constants *J*_1_, *J*_2_, *J*_3_ and *J*_4_ were used in the Hamiltonian, *H* = −2*J*_1_(S_1_S_2_+S_1′_S_2′_) − 2*J*_2_ S_2_S_2′_ − 2*J*_3_(S_1_S_2′_+S_1′_S_2_) − 2*J*_4_(S_1_S_1′_) [35]; they represent the magnetic interactions between Cu1 and Cu2 (or Cu1^i^ and Cu2^i^), Cu2 and Cu2^i^, Cu1 and Cu2^i^ (or Cu1^i^ and Cu2), and between Cu1 and Cu1^i^, respectively. Given the high number of variables, fitting the magnetic data using all four coupling constants led to unreasonable results. Since the distance between Cu1 and Cu2^i^ (or Cu1^i^ and Cu2) and the distance between Cu1 and Cu1^i^ are large enough, *J*_3_ and *J*_4_ could be considered as zero for a simplified model, and better results were thus achieved. The best fitting gave *g* = 2.02, *J*_1_ = −5.1 cm^−1^, *J*_2_ = −172.8 cm^−1^ and *Nα* = 4.8 × 10^−5^ with *R* = 5.5 × 10^−4^ (*R* = Σ[(*χ*_M_)_obs_ − (*χ*_M_)_calc_]^2^/Σ[(*χ*_M_)_obs_]^2^). The large negative value of *J*_2_ reveals that there is a strong antiferromagnetic exchange between the Cu (II) ions mediated by the O_phenolate_ and NO_3_^─^ bridges.

### 2.5. Catalytic Studies

#### 2.5.1. Solvent-Free Microwave (MW) Assisted Oxidation of Secondary Alcohols

**Scheme 2:**
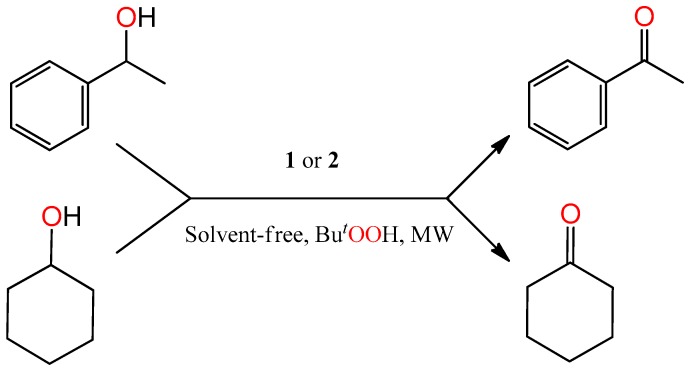
Microwave (MW)-assisted solvent-free oxidation of 1-phenylethanol and cyclohexanol to acetophenone and cyclohexanone, respectively, catalysed by **1** or **2**.

Complexes **1** and **2** were tested as catalyst precursors for the homogeneous oxidation of secondary alcohols [1-phenylethanol (model substrate), cyclohexanol, 2-hexanol and 3-hexanol] to the corresponding ketones following our previously developed procedure [18,31,36,37,38,39] using *tert*-butylhydroperoxide (*t*-BuOOH, Equation (2), used as aq. 70%) as oxidizing agent, under typical conditions of 80–120 °C, microwave (MW) irradiation (5–20 W), 0.5–3 h reaction time and in the absence of any added solvent (Scheme 2 for 1-phenylethanol and cyclohexanol oxidation). Results are summarized in Table 3 and Table 4.

Under typical reaction conditions (120 °C and 0.5 h reaction time) ketone yields up to 37% (TOF = 264 h^−1^) are obtained for the oxidation of 1-phenylethanol (Table 3, entry 3) by the **1**/TBHP/MW system (catalyst/substrate molar ratio of 0.2%) and in the absence of any additive. For a longer reaction time of 1 and 3 h, and for the same catalytic system, the oxidation by TBHP of 1-phenyletanol leads to 75 and 95% of acetophenone, respectively (Table 3, entries 4 and 5). The acetophenone yield value for 1 h reaction (Table 3, entry 4) is significantly higher than that obtained (17%, Table 3, entry 15) without MW assistance, for the same reaction time. Reactions performed at 120 °C with 20 W of MW irradiation and in the presence of complex **2** (**2**/TBHP/MW) resulted only in 28, 42 and 71% of acetophenone after 0.5, 1 and 3 h of oxidation reaction, respectively (Table 3, entries 16–18, respectively). The addition of the heteroaromatic 2-pyrazynecarboxylic acid (Hpca) or of trifluoroacetic acid (TFA) to the **1**/TBHP/MW system [*n* (acid)/*n* (catalyst **1**) = 10], slightly improved the yield from 11 to 14% (Table 3, entry 6) or to 18% (Table 3, entry 9), respectively, when the reaction was performed at 80 °C for 0.5 h.

A major effect was observed in the presence of 2,2,6,6-tetramethylpiperydil-1-oxyl (TEMPO) radical, an efficient promotor in aerobic oxidation of alcohols [11,40,41,42,43,44,45,46,47,48]. In fact, the oxidation of 1-phenylethanol by the **1**/TBHP/TEMPO/MW system afforded substantial increase in the yield of acetophenone achieving 99% after 1 h at 120 °C (Table 3, entry 14) or 88% in only 0.5 h (Table 3, entry 13). Under the oxidation conditions used, the TEMPO additive could be oxidized to the oxoammonium species acting as oxidant, as well as acting as a hydrogen atom abstract from TBHP, enhancing the formation of *tert*-BuOO^•^ and *t*-BuO^•^ [40,41,42,43,44,45,46,47,48]. The **2**/TBHP/TEMPO/MW system was not so effective (Table 3, entries 23 and 24). The blank tests in the absence of metal catalyst (with and without TEMPO) lead to very low conversion (up to 4% yield) of 1-phenylethanol to acetophenone (Table 3, entries 25 and 26).

**Figure 4:**
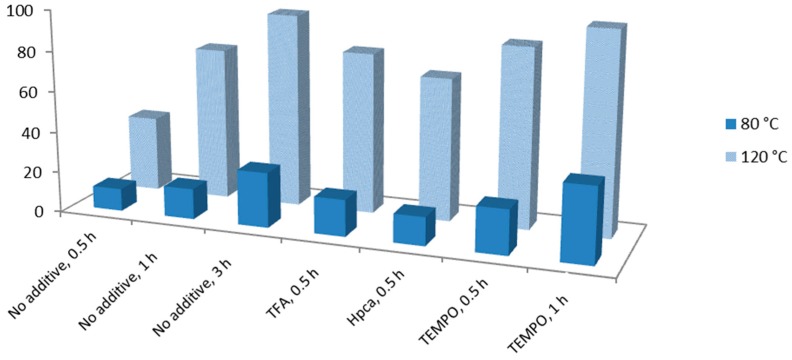
Temperature effect on the yield of acetophenone from MW-assisted peroxidative oxidation of 1-phenylethanol by the **1**/TBHP/MW catalytic system.

Attempts to perform microwave-assisted oxidation of secondary alcohols in the presence of **1** or **2**, in the absence of any additive and at room temperature, failed. Performing the reaction at 80 °C did not allow yields beyond 27% in 3 h (with catalyst **1**), but at 120 °C the product was obtained in 95 (with **1**) or 71% yield (with **2**), in the same reaction time (Table 3, entries 5 and 20). The accelerating effect of the increase in temperature was also observed in the presence of the tested additives (Figure 4). Thus, for example, in the presence of the TEMPO radical, one observes a change in the conversion of 1-phenylethanol from 22 to 88% and in the TOF value from 168 to 1112 h^−1^ (Table 3, entries 12 and 13) when going from 80 or 120 °C, in 0.5 h. This feature also concerns the microwave power since at 80 °C the power went up to 10 W in the first 10 s but then, after reaching the desired temperature, it stabilized at ca. 5 W, while for the temperature of 120 °C, ca. 40 W were reached in the first 10 s followed by stabilisation at 10–15 W for the remaining time.

Complexes **1** and **2** were also tested towards the oxidation of aliphatic alcohols, namely cyclohexanol and the linear 2- and 3-hexanols. As expected, they were less reactive than 1-phenylethanol (benzylic alcohol derivative), leading to moderate yields in the 15–31% range (in 0.5 h reaction at 120 °C, without any additive (Table 4, entries 1, 4, 7, 10, 13 and 16). The efficiency of the oxidation of those alcohols could also be enhanced by using TEMPO radical and the yields increased to 29–58% in 0.5 h (Table 4, entries 2, 5, 8, 11, 14 and 17). Extending the reaction time to 1 h and in the presence of the same promoter, the conversions raised to 87% for cyclohexanol (Table 4, entry 3), 71% for 2-hexanol (Table 4, entry 6) and 59% for 3-hexanol (Table 4, entry 9) with **1** as catalyst. The position (2 or 3) of the OH group in the aliphatic chain of the linear alcohols (2-hexanol and 3-hexanol) appeared not to influence significantly the efficiency of the system, in view of the obtained yields (compare, e.g., entries 4 and 7 or 6 and 9, Table 4, for complex **1**). Blank tests were performed for all aliphatic alcohols in the absence of any catalyst and residual conversions (up to 5%) were recorded.

The catalytic mechanism may proceed through the metal-assisted generation of *t*-BuOO^●^ and *t*-BuO^●^ radicals [40,41,42,43,44,45,46,47,48], upon oxidation or reduction of *t*-BuOOH by a Cu^II^ or Cu^I^ centre (Equations (1) and (2)), respectively, and is summed up in Equations (1)–(6).

Cu(II) + *t*-BuOOH → Cu(I) + *t*-BuOO^•^ + H^+^ (1)

Cu(I) + *t*-BuOOH → Cu(II)-OH + *t*-BuO^•^ (2)

Cu(II)-OH + *t*-BuOOH → Cu(II)-OO-*t*-Bu + H_2_O (3)

*t*-BuO^•^ + R_2_CHOH → *t*-BuOH + R_2_C^•^-OH (4)

*t*-BuOO^•^ + R_2_CHOH → *t*-BuOOH + R_2_C^•^-OH (5)

Cu(II)-OO-*t*-Bu + R_2_C^•^-OH → R_2_C=O + *t*-BuOOH + Cu(I) (6)

#### 2.5.2. Peroxidative Oxidation of Cyclohexane

**Scheme 3:**
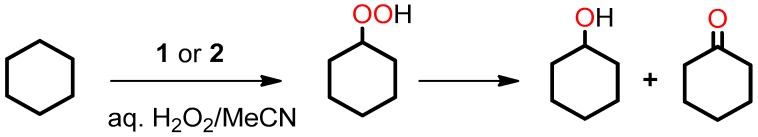
Oxidation of cyclohexane to cyclohexyl hydroperoxide, cyclohexanol and cyclohexanone.

Compounds **1** and **2** were also tested as catalyst precursors in the oxidation of cyclohexane by H_2_O_2_ (Equation (2), used as 50% aqueous solution) at 50 °C in MeCN/H_2_O medium (Scheme 3). The reaction was monitored by Gas Chromatography (GC) to determine the amount of cyclohexanol and cyclohexanone formed, typically after treatment with PPh_3_ (to reduce cyclohexyl hydroperoxide to cyclohexanol) [29,30,49,50,51].

**Figure 5:**
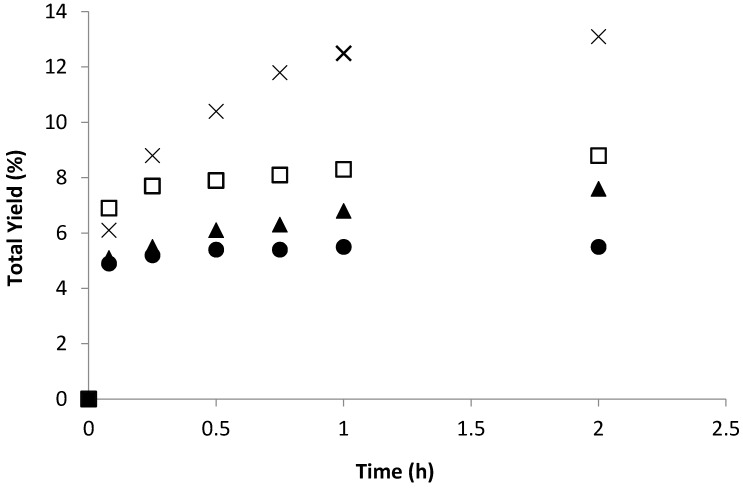
Total yield (cyclohexanol and cyclohexanone) along the time in the oxidation of cyclohexane with aq. H_2_O_2_ catalyzed by **1** in the absence of any additive (□, squares) or in the presence of TFA (1:10) (×, cross), or by **2** in the absence of any additive (●, circles) or in the presence of TFA (1:10) (▲, triangles), at 50 °C in CH_3_CN.

The accumulation of oxygenated products (cyclohexanol and cyclohexanone) in the cyclohexane oxidation catalysed by **1** and **2**, in the absence and in the presence of TFA, is given in Table 5. Both complexes were active for this reaction in the absence of any additive (Figure 5) with approximately 7 and 5% (for **1** and **2**, respectively) of total product yield after ca. 0.5 h (Table 5, entries 3 and 15, for **1** and **2**, respectively). Further increase of the reaction time to 2 h slightly improved the total yield of the products in the presence of **1** and almost had not effect when **2** was the catalyst. The presence of TFA improved the catalytic performance of both copper compounds, more markedly for **1** with the total yield of cyclohexanol and cyclohexanone achieving ca. 13% in 1 h (Table 5, entry 11). The promoting effect of an acid co-catalyst was already observed for other Cu-catalysed systems in the oxidative transformation of alkanes and can be related to (i) its involvement in proton transfer steps, (ii) catalyst activation by unsaturation of the Cu (II) centres upon ligand protonation or to the (iii) facilitation of the formation of peroxo complexes [27,52,53,54,55,56].

The activity exhibited by compounds **1** and **2**, even in the absence of TFA, is higher than that shown, e.g., by [Cu(OTf)_2_(Py_2_S_2_)] (Py_2_S_2_ = 1,6-bis(2′-pyridyl)-2,5-dithiahexane) (with 4.3% overall yield) [57] and is comparable to those of the complexes bearing azathia macrocycles, e.g., [Cu(OTf)_2_(L^3^)] (L^3^ = mixed 14-membered N_2_S_2_ azathia macrocycle) or [Cu(OTf)(L^4^)(H_2_O)](OTf) (L^4^ = nine-membered NS_2_ macrocyclic ligand with a 2-methylpyridyl pendant arm) (overall yield of ca. 8%) [58].

The formation of cyclohexyl hydroperoxide CyOOH was confirmed by GC-MS methods and accounts for a free radical reaction mechanism, which conceivably involves the presence of oxygen-centered radicals, HOO^•^ and HO•, very reactive species, formed upon reaction of the catalyst with hydrogen peroxide (Equations (7) and (8)) [50,59,60,61,62] and enhanced by the presence of TEMPO radical. The HO• radical abstracts hydrogen from cyclohexane CyH to produce cyclohexyl radical Cy• (Equation (9)), which is then trapped by dioxygen to give CyOO• radical (Equation (10)). The latter may react with the oxidant to form CyOOH (Equation (11)). Metal-assisted decomposition of CyOOH to CyO• and CyOO• (Equations (12) and (13)) would then lead to cyclohexanol (CyOH) and cyclohexanone (Cy-H=O) products (Equations (14) and (15)) [61].

Cu(II) + HOOH → HOO• + H^+^ + Cu(I) (7)

Cu(I) + HOOH → HO• + Cu(II) + HO^−^ (8)

HO• + CyH → ROH + Cy•(9)

Cy• + O_2_ → CyOO• (10)

CyOO• + HOOH → CyOOH + HOO•(11)

CyOOH + Cu(I) → CyO• + Cu(II) + HO^−^ (12)

CyOOH + Cu(II) → CyOO• + H^+^ + Cu(I) (13)

CyO• + CyH → CyOH + Cy•(14)

2CyOO• → CyOH + Cy-H=O + O_2_ (15)

The coordination of the oxidant to the metal centre is dependent on the Lewis acidity of the latter. The more Lewis acid metal centre should be easier to reduce. To correlate the electrochemical and catalytic behaviours, the reduction potentials of both complexes were determined by cyclic voltammetry (see above). In fact, complex **1**, the most active one in both oxidation reactions, is easier to reduce than **2** (reduction at a less negative potential, i.e., ^I^*E*_p_^red^ −0.27 V versus −0.43 V vs. SCE).

## 3. Experimental Section

### 3.1. General Materials and Equipment

All synthetic work was performed in air. Commercially available reagents and solvents were used as received, without further purification or drying. Cu(NO_3_)_2_·5H_2_O was used as a metal source for the synthesis of the complexes.

C, H, and N elemental analyses were carried out by the Microanalytical Service of Instituto Superior Técnico. Infrared spectra (4000–400 cm^–1^) were recorded on a Bruker Vertex 70 instrument in KBr pellets; wavenumbers are in cm^–1^. The ^1^H NMR spectra were recorded at room temperature on a Bruker Avance II + 400.13 MHz (UltraShieldTM Magnet, Rheinstetten, Germany) spectrometer. Tetramethylsilane was used as the internal reference and the chemical shifts are reported in ppm. Mass spectra were run in a Varian 500-MS LC Ion Trap Mass Spectrometer equipped with an electrospray (ESI) ion source. For electrospray ionization, the drying gas and flow rate were optimized according to the particular sample with 35 p.s.i. nebulizer pressure. Scanning was performed from *m/z* 100 to 1200 in methanol solution. The compounds were observed in the positive mode (capillary voltage = 80–105 V). The catalytic tests were performed under microwave (MW) irradiation using a focused Anton Paar Monowave 300 microwave fitted with a rotational system and an IR temperature detector, using a 10 mL capacity reaction tube with a 13 mm internal diameter. Gas chromatographic (GC) measurements were carried in a FISONS Instrument GC 8000 series gas chromatograph with a capillary DB-WAX column (30 m × 0.32 mm), a FID detector, helium as the carrier gas and using the Jasco-Borwin v.1.50 software. The magnetic susceptibility measurements were carried out on polycrystalline samples with a Quantum Design MPMS-XL5 SQUID magnetometer in the temperature range of 2–300 K and at an applied field of 2000 Oe. Diamagnetic corrections were estimated from Pascal’s constants for all constituent atoms [63]. The electrochemical experiments were performed in an EG&G PAR 273A potentiostat/galvanostat connected to a personal computer through a GPIB interface.

Syntheses of the pro-ligand H_4_L: The aroylhydrazone Schiff base pro-ligand bis(2-hydroxybenzylidene) isophthalohydrazide (H_4_L) (Scheme 1) was prepared by a reported method [64,65] upon condensation of the 2-hydroxybenzohydrazide with isophthalohydrazide.

Yield: 84%. Anal. Calcd for H_4_L C_22_H_18_N_4_O_4_: C, 65.66; H, 4.51; N, 13.92. Found: C, 65.60; H, 4.48; N, 13.87. IR (KBr pellet, cm^−^^1^): 3212 ν(OH), 3055 ν(NH), 1653 ν(C=O). ^1^H NMR (DMSO-d6, δ): 12.09 (s, 2H, OH), 11.18 (s, 2H, NH), 8.09 (s, 2H, -CH=N), 7.58–6.94 (m, 12H, C_6_H_4_).

Synthesis of [Cu_2_(1κNO^2^:2κN′O^2^-H_2_L)(NO_3_)_2_(H_2_O)_2_] (**1**): 0.402 g (1.0 mmol) of H_4_L was suspended in 25 mL of methanol and 0.525 g Cu(NO_3_)_2_**·**5H_2_O (2.05 mmol) were added. The resultant mixture was stirred at room temperature for 15 min; a dark green solution was obtained. The solution was then filtered and the solvent was allowed to evaporate slowly. After 3–4 days, single crystals suitable for X-ray diffraction were isolated, washed 3 times with cold methanol and dried in open air.

Yield: 0.443 g (66%, with respect to Cu). Anal. Calcd for C_22_H_20_Cu_2_N_6_O_12_: C, 38.43; H, 2.93; N, 12.22. Found: C, 38.36; H, 2.90; N, 12.14. IR (KBr; cm^−^^1^): 3448 ν(OH), 1612 ν(C=N), 1250 ν(C–O)enolic and 1159 ν( N–N). ESI-MS (+): *m/z* 672 [M+H]^+^ (100%).

Synthesis of [Cu_4_(μ-1κNO^2^:2κN′O^2^-H_2_L)_2_(μ-NO_3_)_2_(H_2_O)_4_]**·**2C_2_H_5_OH (**2**): To a 20 mL acetonitrile solution of H_4_L (0.402 g, 1.00 mmol), a 20 mL ethanol solution of Cu(NO_3_)_2_**·**5H_2_O (0.525 g, 2.05 mmol) was added and the reaction mixture was stirred for 20 min at room temperature. The resultant dark green solution was filtered and the filtrate was kept in open air. Dark green single crystals suitable for X-ray diffraction analysis were isolated after 4 days. Crystals were washed 3 times with cold ethanol and dried in open air.

Yield: 0.800 g (64%, with respect to Cu). Anal. Calcd for C_48_H_52_Cu_4_N_10_O_20_ (**2**): C, 42.92; H, 3.90; N, 10.43. Found: C, 42.87; H, 3.84; N, 10.39. IR (KBr; cm^−1^): 3456 ν(OH), 2986 ν(NH), 1609 ν(C=O), and 1168 ν(N–N). ESI-MS (+): *m/z* 1158 [(M-2EtOH)+H]^+^ (100%).

### 3.2. X-Ray Measurements

Good quality single crystals suitable for X-ray diffraction of **1** and **2** were immersed in cryo-oil, mounted in Nylon loops and measured at a temperature of 296 K. Intensity data were collected using a Bruker AXS PHOTON 100 diffractometer with graphite monochromated Mo-Kα (λ 0.71073) radiation. Data were collected using omega scans of 0.5° per frame and full spheres of data were obtained. Cell parameters were retrieved using Bruker SMART [66] software and refined using Bruker SAINT [66] on all the observed reflections. Absorption corrections were applied using SADABS [66]. Structures were solved by direct methods by using SIR97 [67] and refined with SHELXL2014 [68]. Calculations were performed using WinGX version 2014.1 [69]. All non-hydrogen atoms were refined anisotropically. The H-atoms bonded to carbons were included in the model at geometrically calculated positions and refined using a riding model. U_iso_(H) values were defined as 1.2U_eq_ of the parent aromatic and methylene groups and 1.5U_eq_ of the parent methyl ones. The other hydrogen atoms (O–H and N–H) were located in the difference Fourier synthesis and refined, in some cases with the help of distance and angle restraints, their isotropic thermal parameter set at 1.5 times the average thermal parameter of the parent oxygen or nitrogen atom. The structure of **1** appears to have three water molecules per unit cell, but their hydrogen atoms could not be ascertained and the Calc-OH routine of WinGX led to unreasonable locations. The possibility of disordered solvent molecules in a void was taken into consideration and Platon/Squeeze [70] was used to establish a void of 158 Å^3^ with 56 electrons; removing such content in the void did not lead to any improved solution, the reason why such strategy was not followed. Least square refinements with anisotropic thermal motion parameters for all the non-hydrogen atoms and isotropic for the remaining atoms were employed.

### 3.3. Electrochemical Studies

Cyclic voltammetry (CV) and controlled-potential electrolyses (CPE) were undertaken in 0.2 M [*^n^*Bu_4_N][BF_4_]/DMSO electrolyte solutions, saturated with N_2_ before each run, at room temperature. They were performed in a three-electrode cell equipped with a Luggin capillary connected to a silver wire pseudo-reference electrode. Platinum disc (for CV; d = 1 mm) or gauze (CPE) working electrodes were used, as well as platinum wire (CV) or gauze (CPE) counter electrodes. In CPE experiments, the working and counter compartments were separated by a sintered glass frit and the studies were monitored regularly by CV. The redox potentials were measured using ferrocene as internal standard, and their values are quoted relative to the SCE by using the [Fe(η^5^-C_5_H_5_)_2_]^0/+^ redox couple ($E_{1/2}^{OX}$ = 0.44 V vs. SCE for DMSO) [71,72,73,74,75,76].

### 3.4. Typical Procedures and Product Analysis for Catalysis

The microwave-assisted (MW) solvent-free peroxidative catalytic oxidation of 1-phenyethanol was performed in a focused Anton Paar Monowave 300 reactor using a 10 mL capacity cylindric Pyrex tube with a 13 mm internal diameter, fitted with a rotational system and an IR temperature detector. The tube was charged with the alcohol (2.5 mmol), 5 µmol catalyst precursor **1** or **2** (0.2 mol% vs. substrate) and a 70% aqueous solution of *t*-BuOOH (5 mmol), sealed, placed in the microwave reactor and the system left stirring under irradiation (5 or 20 W) at 80 or 120 °C for 0.5–3 h. After cooling to room temperature, 150 µL of benzaldehyde (internal standard) and 2.5 mL of MeCN (to extract the substrate and the organic products from the reaction mixture) were added. The obtained mixture was stirred for 10 min and then a sample (1 µL) was taken from the organic phase and analysed by GC.

The peroxidative oxidations of cyclohexane were performed in round bottom flasks with vigorous stirring and using MeCN as solvent (up to 5.0 mL total volume), under air. The catalyst precursors **1** and **2** and trifluoroacetic acid (TFA, optional), in the form of a stock solution in acetonitrile, were mixed, cyclohexane (0.25 mL, 2.3 mmol) was included and the reaction started when hydrogen peroxide was added (50% in H_2_O, 0.68 mL, 11 mmol). The concentrations of the reactants were thus as follows: catalyst precursor (5 × 10^−4^ mol L^−1^), TFA (0.005 mol L^−1^), substrate (0.46 mol L^−1^) and H_2_O_2_ (2.2 mol L^−1^). The reaction mixture was stirred at 50 °C for 2 h and at 5, 15, 30, 45, 60 and 120 min, an aliquot was taken and analysed by GC using nitromethane (50 µL) as internal standard. Before the GC analysis an excess of triphenylphosphine was added, in order to reduce the formed cyclohexyl hydroperoxide to the corresponding alcohol and hydrogen peroxide to water, following a method developed by Shul’pin [49,50,51]. Blank experiments were performed and confirmed that no product of cyclohexane oxidation was obtained unless the metal catalyst was used.

For the GC measurements, the temperature of injection was 240 °C. The initial temperature of the column was maintained at 100 °C (oxidation of cyclohexane) or 120 °C (oxidation of alcohol) for 1 min, then raised 10 °C/min up to 180 °C (oxidation of cyclohexane) or 200 °C (oxidation of alcohol), and held at this temperature for 1 min. Attribution of peaks was made by comparison with chromatograms of genuine samples and, in some cases, by GC-MS analyses using a Perkin Elmer Clarus 600 C instrument (He as the carrier gas), equipped with a 30 m × 0.22 mm × 25 μm BPX5 (SGE) capillary column.

## 4. Conclusions

The dinuclear [Cu_2_(1κ*NO*^2^:2κ*N′O′*^2^-H_2_L)(NO_3_)_2_(H_2_O)_2_] (**1**) and the tetranuclear [Cu_4_(μ-1κ*NO*^2^:2κ*N′O′*^2^-HL)_2_(μ-NO_3_)_2_(H_2_O)_4_] (**2**) complexes derived from bis(2-hydroxybenzylidene)isophthalohydrazide (H_4_L) have been synthesized and characterized. The electrochemical studies show two one-electron cathodic reductions [Cu^II^ → Cu^I^ and Cu^I^ → Cu^0^]. The variable temperature magnetic susceptibility measurements of **2** show strong antiferromagnetic exchange between the Cu (II) ions meadiated by the O-phenolate and NO_3_^─^ bridges.

Both complexes act as catalyst precursors towards the solvent-free microwave-assisted oxidation of alcohols and the peroxidative oxidation of alkanes under mild conditions. The highest activity and selectivity with a maximum product yield of 99% (for the 1-phenylethanol oxidation after 1 h without any additive) were observed with **1**. In the case of oxidation of cyclohexane, **1** also exhibits a better activity (a maximum product yield of 13% after 3h) than **2**.

The catalytic oxidations under microwave assisted and solvent-free conditions have significance for the development of environmentally friendly catalytic systems and deserve further investigations. Our study was an attempt to contribute to this aim.

## Figures and Tables

**Table 1:** Crystal data and structure refinement details for complexes **1** and **2**.

	1	2·2EtOH
Empirical formula	C_22_H_20_Cu_2_N_6_O_15_	C_48_H_50_Cu_4_N_10_O_20_
Formula Weight	735.52	1341.14
Crystal system	Triclinic	Triclinic
Space group	*P*−1	*P*−1
Temperature/K	296 (2)	296 (2)
*a*/Å	7.8618 (4)	9.5722 (5)
*b*/Å	10.1197 (6)	10.0444 (5)
*c*/Å	18.1173 (10)	14.9699 (8)
α/°	102.605 (3)	71.735 (2)
*β/°*	98.203 (2)	76.323 (3)
γ/°	90.384 (3)	80.058 (3)
*V* (Å^3^)	1391.19 (13)	1320.44 (12)
*Z*	2	1
D_calc_ (g cm^–3^)	1.756	1.687
*μ* (Mo K*α*) (mm^–1^)	1.615	1.677
Rfls. collected/unique/observed	15,443/4623/2087	17,480/4694/2675
*R*_int_	0.1339	0.0913
Final *R*1 ^*a*^, *wR*2 ^*b*^ (*I ≥* 2*σ*)	0.0718, 0.1441	0.0607, 0.1326
Goodness-of-fit on *F*^2^	0.996	1.039

***^a^*** R = Σ||*F_o_*|–|*F_c_*||/Σ|*F_o_*|; ^*b*^ wR (F^2^) = [Σw(|F_o_|^2^ – |F_c_|^2^)^2^/Σw|F_o_|^4^]^½^.

**Table 2:** Selected bond distances (Å) and angles (º) in complexes **1–2**.

Parameter	1	2
Cu_(water)_-O_phenolate_	1.881(6)	1.903(4)
Cu_(nitrate)_-O_phenolate_	1.891(5)	1.925(4)
Cu_(water)_-O_ketone_	1.964(6)	2.017(4)
Cu_(nitrate)_-O_ketone_	1.984(5)	1.919(4)
Cu-O_nitrate_	1.961(7) 2.427(7) 2.69(1)	2.680(7) 2.709(5)
Cu-O_water_	2.019(8) 2.131(8)	1.970(4) 2.251(5)
Cu-N	1.935(7) 1.938(6)	1.905(5) 1.933(5)
N-N	1.379(8) 1.399(9)	1.373(7) 1.405(6)
Shortest		8.457 [Cu_(water)_···Cu_(nitrate)_]
intramolecular Cu···Cu	9.471 [Cu_(water)_···Cu_(nitrate)_]	2.928 [Cu_(nitrate)_···Cu_(nitrate)_]
intermolecular Cu···Cu	5.471 [Cu_(water)_···Cu_(nitrate)_]	6.120 [Cu_(water)_···Cu_(nitrate)_]
Largest		
O-Cu_(water)_-O	172.3(3)	170.53(18)
O-Cu_(water)_-N	149.8(5)	167.0(2)
O-Cu_(nitrate)_-O	171.6(2)	174.01(18)
O-Cu_(nitrate)_-N	176.5(3)	170.71(19)

**Table 3:** Selected results for the MW-assisted oxidation of 1-phenylethanol by *tert*-butylhydroperoxide (TBHP) with **1** and **2** as catalyst precursors *^a^*.

Entry	Catalyst Precursor	Reaction Time (h)	Temperature (°C)	Additive (μmol)	Yield *^b^* (%)	TOF *^c^* (h^−1^)
1	1	0.5	80	-	11	108
2		3	80	-	27	74
3		0.5	120	-	37	264
4		1	120	-	75	494
5		3	120	-	95	222
6		0.5	80	Hpca (50)	14	118
7		0.5	120	Hpca (50)	70	538
8		0.5	80	Hpca (100)	5	64
9		0.5	80	TFA (50)	18	182
10		0.5	120	TFA (50)	79	501
11		0.5	80	TFA (100)	6	89
12		0.5	80	TEMPO (30)	22	168
13		0.5	120	TEMPO (30)	88	1112
14		1	120	TEMPO (30)	99	755
15 *^d^*		1	120	-	17	111
16		3	120	-	42	98
17	2	0.5	80	-	5	56
18		0.5	120	-	28	182
19		1	120	-	42	212
20		3	120	-	71	137
21		0.5	80	TFA (50)	8	82
22		0.5	80	Hpca (50)	6	67
23		0.5	80	TEMPO (30)	10	78
24		0.5	120	TEMPO (30)	46	237
25	None	1	120	-	1	6
26	None	1	120	TEMPO	4	21

***^a^*** Reaction conditions (unless stated otherwise): 2.5 mmol of substrate, 5 μmol of **1** or **2** (0.2 mol% vs. substrate)**,** 5 mmol of *t*-BuOOH (aq. 70%), 80 or 120 °C, microwave irradiation (5–20 W). ***^b^*** Molar yield (%) based on substrate, i.e., moles of product per 100 mol of substrate determined by G.C. ***^c^*** Turnover frequency = number of moles of product per mol of metal catalyst per hour (turnover number per hour). *^d^* Under conventional heating.

**Table 4:** MW-assisted oxidation of selected alcohols by TBHP with **1** and **2** as catalyst precursors *^a^*.

Entry	Catalyst Precursor	Substrate	Reaction Time (h)	Additive (μmol)	Yield *^b^* (%)	TOF *^c^* (h^−1^)
1	1	cyclohexanol	0.5	-	31	380
2			0.5	TEMPO (30)	58	568
3			1	TEMPO (30)	87	471
4		2-hexanol	0.5	-	21	204
5			0.5	TEMPO (30)	45	426
6			1	TEMPO (30)	71	726
7		3-hexanol	0.5	-	20	194
8			0.5	TEMPO (30)	43	398
9			1	TEMPO (30)	69	321
10	2	cyclohexanol	0.5	-	22	222
11			0.5	TEMPO (30)	34	382
12			1	TEMPO (30)	59	285
13		2-hexanol	0.5	-	17	176
14			0.5	TEMPO (30)	31	368
15			1	TEMPO (30)	49	225
16		3-hexanol	0.5	-	15	168
17			0.5	TEMPO (30)	29	358
18			1	TEMPO (30)	51	255

***^a^*** Reaction conditions (unless stated otherwise): 2.5 mmol of substrate, 5 μmol of **1** or **2** (0.2 mol% vs. substrate), 5 mmol of *t-*BuOOH (aq. 70%), 120 °C, microwave irradiation (20 W power). *^b^* Molar yield (%) based on substrate, i.e., moles of product per 100 mol of substrate determined by GC. *^c^* Turnover frequency = number of moles of product per mol of metal catalyst per hour (turnover number per hour).

**Table 5:** Total yield (cyclohexanol and cyclohexanone) with time in the oxidation of cyclohexane by H_2_O_2_ at 50 °C in CH_3_CN catalysed by **1** or **2** *^a^*.

Entry	Precatalyst	Acid Co-Catalyst	Reaction Time (min)	Yield (%)		
				OL	ONE	Total *^b^*
1	1	-	5	3.7	3.2	6.9
2		-	15	4.1	3.6	7.7
3		-	30	4.2	3.7	7.9
4		-	45	4.3	3.8	8.1
5		-	60	4.5	3.8	8.3
6		-	120	4.8	3.9	8.7
7		TFA	5	3.8	2.3	6.1
8		TFA	15	5.3	3.5	8.8
9		TFA	30	5.9	4.5	10.4
10		TFA	45	6.6	5.2	11.8
11		TFA	60	7.2	5.3	12.5
12		TFA	120	7.8	5.3	13.1
13	2	-	5	3.3	1.6	4.9
14		-	15	3.5	1.7	5.2
15		-	30	3.5	1.9	5.4
16		-	45	3.6	1.8	5.4
17		-	60	3.7	1.8	5.5
18		-	120	3.9	1.6	5.5
18		TFA	5	2.9	2.2	5.1
20		TFA	15	3.6	1.9	5.5
21		TFA	30	4.1	2.0	6.1
22		TFA	45	4.4	1.9	6.3
23		TFA	60	4.8	2.0	6.8
24		TFA	120	5.1	2.5	7.6

***^a^*** Reaction conditions: C_6_H_12_ (0.46 M), **1** or **2** (5 × 10^−4^ M), TFA (5 × 10^−^^3^ M), H_2_O_2_ (50% aq, 2.2 M), MeCN (up to 5 mL total volume), 50 °C. *^b^* Moles of products [cyclohexanol (OL) + cyclohexyl hydroperoxide (OOH) + cyclohexanone (ONE)]/100 mol of C_6_H_12_, determined by GC after treatment with PPh_3_. *^c^* Moles of products (cyclohexanol + cyclohexanone) per mol of catalyst precursor.

## Supplementary Materials

The following are available online, Cif: Compound **1** and Compound **2**. CCDC 1858873 and 1858872 for **1** and **2** contain the supplementary crystallographic data for this paper. This data can be obtained free of charge from The Cambridge Crystallographic Data Centre via www.ccdc.cam.ac.uk/data_request/cif.
